# Corrosion Performance of Nano-TiO_2_-Modified Concrete under a Dry–Wet Sulfate Environment

**DOI:** 10.3390/ma14195900

**Published:** 2021-10-08

**Authors:** Chao Xu, Hao-Hao Liao, You-Liang Chen, Xi Du, Bin Peng, Tomas Manuel Fernandez-Steeger

**Affiliations:** 1 Department of Civil Engineering, University of Shanghai for Science and Technology, 516 Jungong Road, Shanghai 200093, China; chao.xu@rwth-aachen.de (C.X.); 192591781@st.usst.edu.cn (H.-H.L.); xi.du@unsw.edu.au (X.D.); BinPeng@usst.edu.cn (B.P.); 2 School of Civil and Environmental Engineering, The University of New South Wales, Sydney, NSW 2052, Australia; 3 Department of Engineering Geology, Institute of Applied Geosciences, Faculty VI Planning Building Environment, Technische Universität Berlin, 10587 Berlin, Germany; fernandez-steeger@tu-berlin.de

**Keywords:** nano, dry–wet cycle, compressive strength, PFC2D

## Abstract

This study compared the effects of the sulfate dry–wet cycle on the properties of ordinary concrete and nano-TiO_2_-modified concrete, including the mass loss rate, ultrasonic wave velocity, compressive strength, and XRD characteristics. In addition, a series of compression simulations carried out using the PFC2D software are also presented for comparison. The results show the following: (1) with an increase in dry–wet cycles, the damage to the concrete gradually increased, and adding nano-TiO_2_ into ordinary concrete can improve the material’s sulfate resistance; (2) after 50 sulfate dry–wet cycles, the mass loss rate of ordinary concrete was –3.744%, while that of nano-TiO_2_-modified concrete was −1.363%; (3) the compressive strength of ordinary concrete was reduced from 41.53 to 25.12 MPa (a reduction of 39.51%), but the compressive strength of nano-TiO_2_-modified concrete was reduced from 49.91 to 32.12 MPa (a reduction of 35.64%); (4) after a sulfate dry–wet cycle, the nano-TiO_2_-modified concrete surface produced white crystalline products, considered to be ettringite based on the XRD analysis; (5) when considering the peak stress and strain of the concrete samples, the numerical results agreed well with the test results, indicating the reliability of the method.

## 1. Introduction

In recent years, there has been an increase in the number of construction projects worldwide; as a result, the use of concrete materials has increased year on year. Due to the influence of the environment, concrete is inevitably influenced by sulfate in nature; in particular, the influence is more significant in coastal areas. Normally, solid salts cannot erode concrete; however, when it exists in the form of a solution, it reacts with concrete, which can not only cause harmful expansion and cracking, but can also reduce the bond strength between the cement slurry itself and the aggregate [1,2,3,4]. At present, the main measures used are mixed cement containing slag or pozzolan to reduce Ca(OH)_2_ in hardened cement slurry. Pozzolan can react with Ca(OH)_2_, so that there is not enough Ca(OH)_2_ in the paste to react with sulfate [5,6,7]. In addition, high-pressure steam curing of concrete can improve its resistance to sulfuric acid corrosion, which makes the hydration product C_3_AH_6_ more stable and promotes the reaction of Ca(OH)_2_ with silica [8,9]. Moreover, corrosion inhibitors are widely used. Inhibitors prevent the onset of corrosion by increasing the pH of concrete or by reducing harmful ions that can cause the corrosion of concrete [10]. The frequently used corrosion inhibitors include inorganic nitrites [11], inorganic phosphate and molybdenum, monofluorophosphate, and organic inhibitors such as alkanolamine and amine groups [12,13]. The above methods all reduce the content of sulfate in concrete, thereby reducing the corrosion of sulfate in concrete. On the contrary, by increasing the permeability of concrete, the corrosion of concrete caused by external sulfate can be reduced. The permeability of concrete is mainly determined by the microstructure of hardened cement paste.

A large number of studies have shown that nanomaterials can improve the microstructure and increase the impermeability of concrete [14,15], among which nano-silica is the most widely studied nano-additive related to cement-based materials. The material can be delivered in powdered or liquid form. At the same time, due to its extremely small-sized particles, nano-silica can fill the voids between cement particles, leading to a higher packing-level “filling effect” and also generating a denser binding matrix with more calcium silicate hydrate. Consequently, a significant improvement in both durability and mechanical properties can be obtained [16,17]. Saloma et al. [18] concluded that the sulfate resistance of nano-silica-based concrete is much better than that of plain concrete. However, there are few studies on the mixing of TiO_2_ particles in concrete. Thus, this article, through a nano-TiO_2_ concrete experiment, mainly discusses its influence on the resistance of concrete to sulfuric acid attack.

At present, the finite element, block discrete element, and particle flow methods are frequently used to analyze the mechanical properties of concrete structures. The finite element method is suitable for the analysis of deformable media, yet it cannot reflect the complex interaction between particles and highly nonlinear behavior and cannot describe the fluid deformation characteristics of bulk materials [19]. The finite element method is not ideal for solving problems such as large deformation, discontinuity, infinite domain, or stress concentration either. The block discrete element method is suitable for dealing with the failure problem, in which all nonlinear deformation and failures are concentrated on the joint surface, but it has certain limitations for continuous media, so the determination of the normal parameters on the joint surface still needs to be solved [20]. In recent years, the particle flow method has become increasingly applied in the analyses of a large number of materials. The particle flow method is not limited by deformation and can easily deal with the mechanical problems of discontinuous media. In particular, the method can deal with the cumulative damage and failure mechanism of micro-media under complex conditions, which reflects the different physical relations of multi-image media [21]. Particle flow code (PFC), which was applied in this paper, is a software-based particle flow method. Compared with the fine element, PFC can more truly reflect the influence of nanoparticles on the compressive properties of concrete. Generally, researchers only use one contact model to reflect the mechanical properties of concrete. In this paper, two contact models (a particle softening model) were set up to reflect the effect of dry–wet cycles on concrete erosion from a microscopic level. The compressive failure of concrete is brittle, and the instant failure of the specimen makes it difficult to obtain many properties. The parameters of concrete are found in this paper, when considering the peak stress and strain of the concrete samples, the numerical results agreed well with the test results, indicating the reliability of micro parameters, this has laid a foundation for the future research of nano-TiO_2_-modified concrete by PFC. Some properties of concrete can be predicted by the simulated specimen, such as how many tensile cracks and shear cracks are produced in the failure.

In general, the main subject of this paper is the influence of nano-TiO_2_ on the resistance of concrete to sulfuric acid attack, including mass loss rate, ultrasonic wave velocity, compressive strength, and XRD analyses. The compression simulation of ordinary and nano-TiO_2_ concrete was carried out by PFC2D software. The micro parameters of concrete in PFC software are obtained. When considering the peak stress and strain of the concrete samples, the numerical results agreed well with the test results, indicating the reliability of the method.

## 2. Materials and Methods

### 2.1. Materials

The raw materials included gravel, cement, sand, nano-TiO_2_, sulfate, water-reducing agent, and water. The cement was a conch brand, PO42.5 grade, plain Portland cement; its relative density was 3.12 g/cm^3^. The main chemical components of the cement are shown in Table 1. Natural sand was selected with an aperture of 0.6 mm. The particle size distribution was measured by a laser particle size analyzer. The maximum particle size of the sand was less than 1.2 mm, and most of the particles (above 99%) were below 900 μm; more than 50% of the particles were less than 500 μm, and the average particle size was 0.46 mm. The particle size of the limestone crushed stone was 5–20 mm of continuous gradation, the crushing index was 4.8%, and the surface density was 2700 kg/m^3^. The anhydrous sodium sulfate was a white powder with hygroscopicity, easily to dissolved in water, and the aqueous solution was neutral. The water reducer was made of BASF 2651F, which was a modified polycarboxylic ether made by the spray-drying process. It had good fluidity, a uniform color, and an obvious increase in strength. The titanium dioxide was white; the details of nano-TiO_2_ are shown in Table 2.

### 2.2. Specimen Preparation

To begin, some exploratory tests were carried out and it was found that nano-TiO_2_ has strong water absorption. When the content nano-TiO_2_ reaches 5%, the concrete is difficult to mix and form, so the use of a water reducer was necessary. A water reducer ensures that the nano-TiO_2_-modified concrete becomes a test specimen. In order to reduce the influence of the water reducer on the test results, the two groups used the same 4.1 g amount of water reducer, and nano-TiO_2_ accounted for 3% of cement in this paper (see Table 3 for specific mix coordination). According to the mix proportion in Table 3, the nanomaterials, water reducer, and water were weighed and stirred to prepare the nano-TiO_2_ solution. To ensure that the nanoparticles were fully dispersed in the aggregate, the precipitation of the nanoparticles in the solution should be prevented and the nanoparticles should be evenly dispersed in the aggregate, which is key to the preparation of nano-modified concrete. The gravel, cement, and sand were weighed and poured into a 5 L cement mortar mixer and slowly dry-stirred for 2–4 min. After the aggregate was fully mixed, the prepared nano-TiO_2_ solution was added to the aggregate, and then the remaining two thirds of the water was gradually added and rapidly mixed for 3–5 min. When the fluidity met the requirements (the paste did not agglomerate in appearance and had certain workability), the mixing procedure was completed to put the mixture into the mold for molding. The size of the compression specimen was 100 mm× 100 mm× 100 mm.

### 2.3. Dry–Wet Cycle Steps

**Figure 1:**
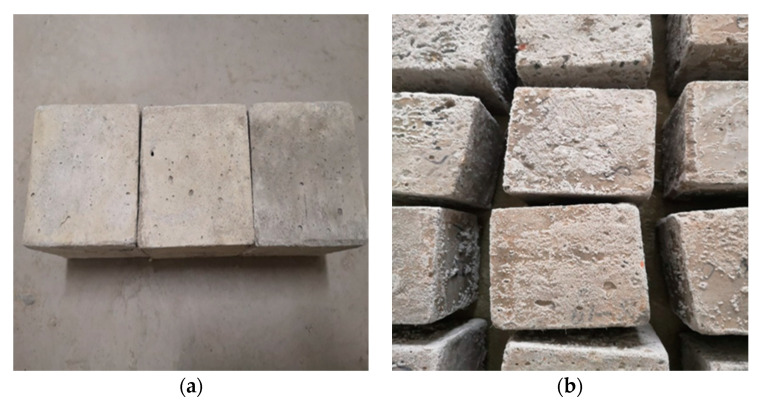
Nano-TiO_2_ modified concrete specimen. (**a**) Specimen at room temperature. (**b**) Specimen after dry–wet cycle.

The specimens were cured for 28 days after the preparation. Then, their surfaces were wiped clean with a wet cloth. Next, the specimens were put into an 80 °C dryer and dried for 2 h. After drying, the specimens were numbered and weighed, and their initial quality and ultrasonic wave velocity were recorded. Thereafter, the samples were put into the sulfate dry–wet cycle testing machine. At the same time, the 5% sodium sulfate solution was injected into the machine until the liquid level was 5 mm higher than the top of samples. The setting of the dry–wet cycle machine one stage every 10 dry–wet, with each cycle lasting 24 h, in which the sulfate soaking time was 15 h and the temperature stage was 30 °C. The drying time was 6 h and the temperature was 80 °C. After 20, 30, 40, and 50 dry–wet cycles, the mass loss rate and ultrasonic wave velocity of each stage were calculated. The surface of the nano-TiO_2_-modified concrete after the dry–wet cycles is shown in Figure 1. Obviously, the surface of the nano-modified concrete was flat and clean, but after sulfuric acid corrosion, a white powder was produced. During the curing stage of the specimen, the water molecules in the concrete specimen evaporated, the concentration of sodium sulfate solution increased, and the crystallization of sodium sulfate occurred. The macroscopic concrete surface is covered with a layer of white crystalline products.

## 3. Results and Discussion

### 3.1. Mass Loss Rate

**Figure 2:**
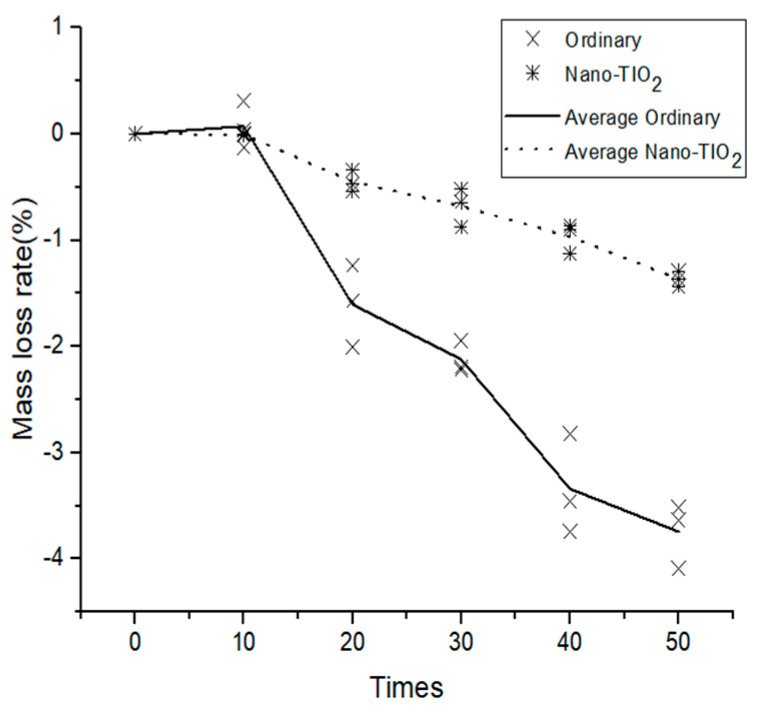
The influence of dry–wet cycles on the concrete mass-loss rate.

The mass loss rate of the specimen after the dry–wet cycle is shown in Figure 2. In general, with an increase in the dry–wet cycle, the mass loss rate of the ordinary concrete increased negatively. At 10–20 and 30–40 dry–wet cycles, the negative growth of the concrete mass loss rate was the fastest. With an increase in the number of dry–wet cycles, the mass loss rate of the nano-TiO_2_-modified concrete experienced a small positive increase of 0.072% at 10 cycles and then gradually decreased. Because fine cracks appeared on the surface of the nano-TiO_2_-modified concrete at 10 cycles, which led to the growth of mass loss, with a further increase in dry–wet cycles, the fine cracks gradually expanded and the sulfate solution entered the surface of the specimen to produce a crystalline powder, which resulted in a decrease in the mass loss rate of the specimen. After 50 dry–wet cycles, the mass loss rate of the ordinary concrete was −3.744%, while the mass loss rate of nano-TiO_2_-modified concrete was −1.363%. There were few crystalline products in the cracks of the nano-TiO_2_-modified concrete, which confirms that nano-TiO_2_ improves the impermeability of concrete and reduces the gap products.

### 3.2. Ultrasonic Wave Velocity

**Figure 3:**
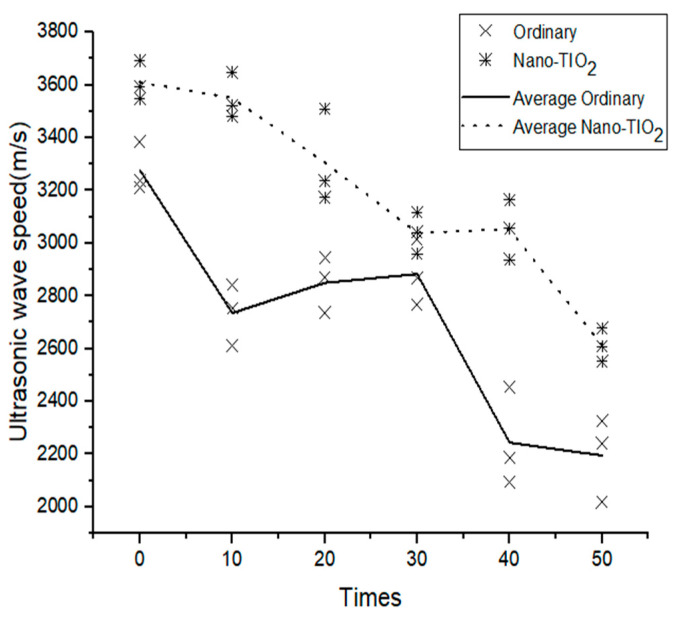
The influence of dry–wet cycles on the concrete ultrasonic wave velocity.

The ultrasonic wave velocity of the specimen after dry–wet cycles is shown in Figure 3. It can be seen that the ultrasonic wave velocity of the ordinary concrete was 3277 m/s at the beginning and 2735 m/s after 10 dry–wet cycles, which indicates that sulfate damaged the specimen. However, the ultrasonic wave velocity of the ordinary concrete increased slightly at 10–30 dry–wet cycles. Thus, it can be considered that cement slurry continues to be hydrated, and sulfate infiltrating crystallization can fill the cracks in concrete, which results in a small increase in the ultrasonic wave velocity of ordinary concrete at 10–30 dry–wet cycles. After 50 dry–wet cycles, the ultrasonic wave velocity of the ordinary concrete was 2195 m/s (33.02% lower than that of the initial time). The ultrasonic wave velocity of the nano-TiO_2_-modified concrete was 3610 m/s at the beginning. From 0 to 30 dry–wet cycles, the ultrasonic wave velocity of the nano-TiO_2_-modified concrete was always in a declining stage, which confirms that the nano-TiO_2_-modified concrete had been eroded by sulfate. In the 30–40 dry–wet cycles, the ultrasonic wave velocity of the nano-TiO_2_-modified concrete remained stable. After 50 dry–wet cycles, the ultrasonic wave velocity of the nano-TiO_2_-modified concrete was 2612 m/s (27.65% lower than that of the initial time).

### 3.3. Compressive Strength

**Figure 4:**
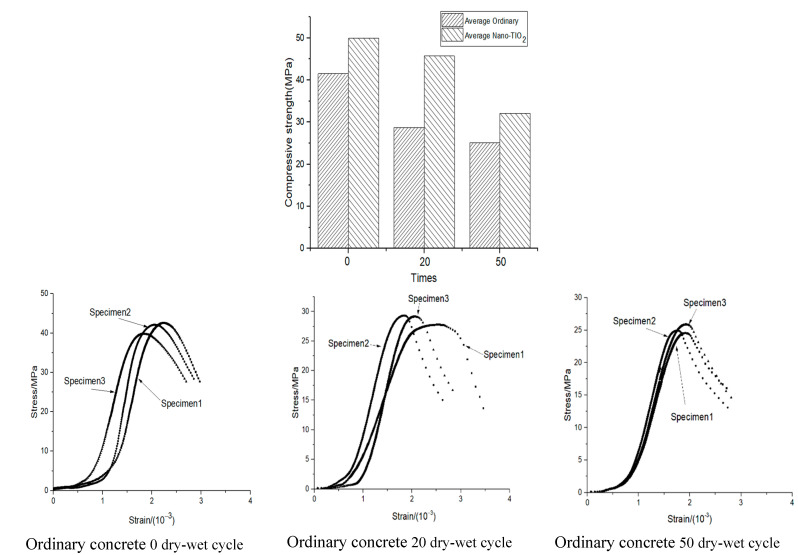

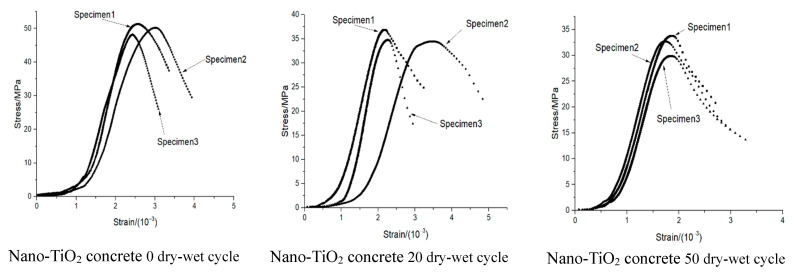
The influence of dry–wet cycles on the concrete compressive strength.

The compressive strength of the specimen after the dry–wet cycles is shown in Figure 4. It can be seen that the average maximum compressive strength of the ordinary and nano-TiO_2_-modified concrete decreased with the increase in dry–wet cycles. In the beginning, the maximum average compressive strength of the ordinary and nano-TiO_2_-modified concrete was 41.53 and 49.91 MPa, respectively. The average maximum compressive strength of the concrete with nanoparticles increased by 20.17%. After 50 dry–wet cycles, the compressive strength of the ordinary concrete decreased by 39.51% (from 41.53 to 25.12 MPa), while the compressive strength of the nano-TiO_2_-modified concrete decreased by 35.64% (from 49.91 to 32.12 MPa).

The stress–strain of the specimen after the dry–wet cycles is shown in Figure 4. It can be seen that the stress–strain of the concrete can be divided into three stages, the first stage being the compaction stage, the second stage the elastic stage, and the third stage the yield stage. Compared to the ordinary concrete, the corresponding strain of the nano-TiO_2_-modified concrete at peak stress increased by approximately 0.5 × 10^−3^. After 50 dry–wet cycles, the strain corresponding to the peak stress of the ordinary and nano-TiO_2_-modified concrete decreased by approximately 1.9 × 10^−3^. The influence of the dry–wet cycles on the compaction and elastic stages was small. The stress–strain trend of the ordinary and nano-TiO_2_-modified concrete was similar. However, in the yield stage, it had a certain influence: At 20 dry–wet cycles, the specimens showed dispersion after the peak, and the ductility of some of the specimens increased.

### 3.4. XRD

**Figure 5:**
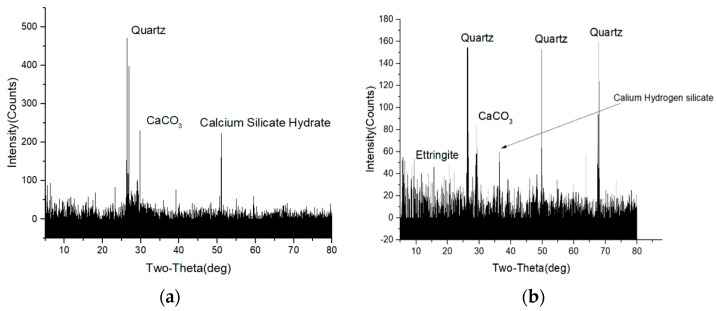
Powder diffraction patterns from concrete. (**a**) Nano-TiO_2_ modified concrete at room temperature. (**b**) Nano-TiO_2_ modified concrete after dry-wet cycle.

Figure 5 compares the XRD analysis of the nano-TiO_2_-modified concrete under normal temperature curing and after the dry–wet cycles. As can be seen, there are several diffraction peaks in the nano-TiO_2_-modified concrete cured at room temperature: After comparing and analyzing the corresponding characteristic angles, quartz, calcium carbonate, calcium silicate hydrate, etc., were found. There were quartz, calcium carbonate, calcium silicate hydrate, and Ettringite in the spectrum of the nano-TiO_2_-modified concrete after the dry–wet cycles. Quartz is the main component of sand, calcium carbonate comes from the limestone and the carbonation reaction in the environment, hydrated calcium silicate is the hydration product of cement slurry, and ettringite is the product of sulfate attack. Sulfate attack is a complex physical–chemical process, and it is generally believed that the process of sulfate attack on concrete consists of the following five main chemical reactions 22:

(1) $${\mathrm{Na}}_{2}{\mathrm{SO}}_{4}\;+\;2H+\mathrm{CH}\;≜\;C\bar{S}H_{2}\;+\;2\mathrm{NaOH}$$

(2) $$C_{4}A\bar{S}H_{12}\;+\;2C\bar{S}H_{2}\;+\;16H\;≜\;C_{6}{\mathrm{AS}}_{3}H_{32}$$

(3) $$3C_{4}A\bar{S}H_{12}\;+\;3\mathrm{Na}{\mathrm{SO}}_{4}\;+\;34H\;≜\;6\mathrm{NaOH}\;+\;2\mathrm{Al}{\left(\mathrm{OH}\right)}_{3}\;+\;2C_{6}A\bar{S}H_{32}$$

(4) $$C_{3}A\;+\;3C\bar{S}H_{2}\;+\;26H\;≜\;C_{6}A\bar{S}H_{32}$$

(5) $$C_{3}{\mathrm{AH}}_{6}\;+\;3C\bar{S}H_{2}\;+\;20H\;≜\;C_{6}A\bar{S}H_{32}$$

where H, C, A, and S denote H_2_O, CaO, Al_2_O_3_, and SO_3_, respectively. Products such as ettringite and dihydrate gypsum expand by absorbing water, which destroys the micro-housekeeper structure of concrete.

## 4. Compression Simulation

### 4.1. Generation and Servo of Compression Specimens

**Figure 6:**
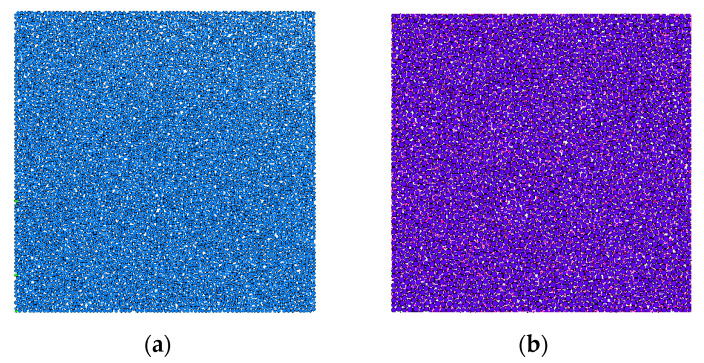
Simulation diagram of compression specimens. (**a**) Ordinary concrete. (**b**) Nano-TiO_2_ modified concrete.

PFC2D software was used for modeling. The calculation unit of the aggregate was spherical particles. The size of the specimen was 100 mm × 100 mm, where the ordinary concrete produced 7080 particles, as shown in Figure 6a, and the nano-TiO_2_-modified concrete produced 10,502 particles, as shown in Figure 6b. It can be seen that many white voids occurred in the ordinary concrete, while the voids in the nano-TiO_2_-modified concrete were filled with a large number of red nanoparticles. When the initial model was generated, the contact accuracy in the model system was not great enough, and there was an interaction between particles. Thus, the initial model was unable to simulate the specimen well, which could be solved by the servo mechanism. When the ratio of the average unbalance force to the average contact force was less than 1 × 10^–5^, the servo system was stopped, and the specimen state reached stability.

### 4.2. Microscopic Parameters of the Specimens

**Figure 7:**
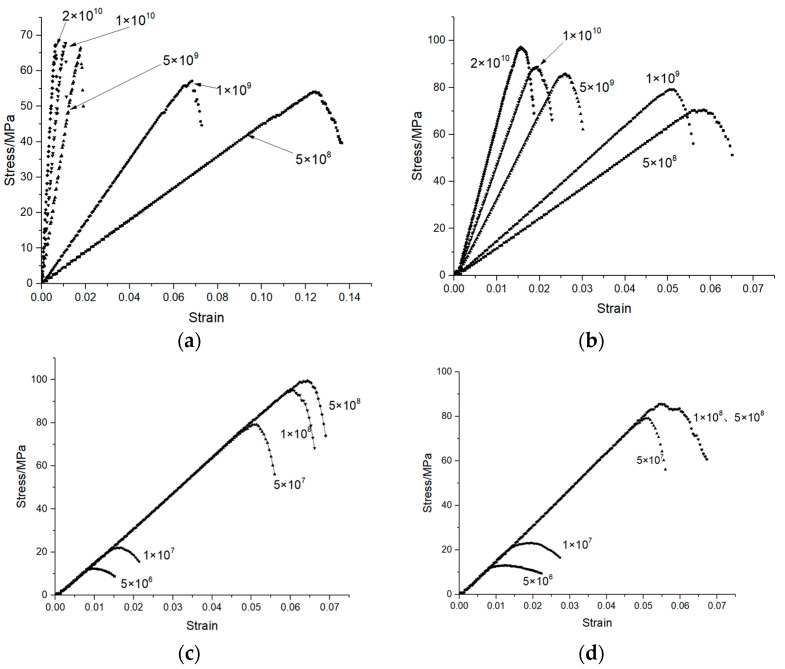
The influence of four main parameters on compressive stress–strain. (**a**) Pb_emod. (**b**) Cb_emod. (**c**) Ten. (**d**) Coh.

**Figure 8:**
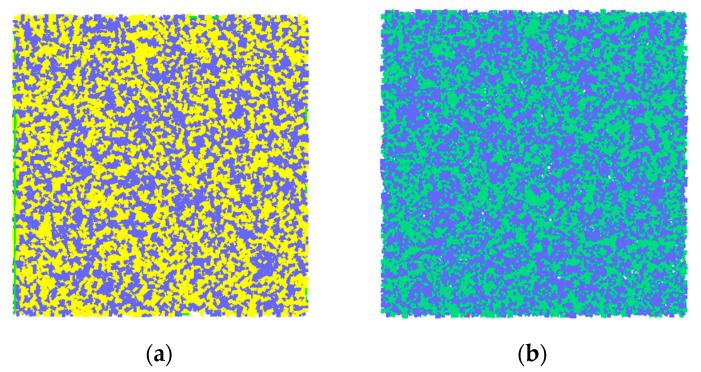
Simulation contact of specimens. (**a**) Ordinary concrete. (**b**) Nano-TiO_2_ modified concrete.

The parallel bonding model was used in the simulation. The contact force between the particles came from the contact force generated by the overlapping of particles and from the bonding force and torque generated by the parallel bonding model. When bonded, it can resist torque and become linear elasticity until the force exceeds the strength limit and the bonding model is destroyed. Due to the dry–wet cycles, some of the particles became soft, and two groups of parallel bonding combinations were used to reflect the softening phenomenon: First, the influence of four main parameters on compressive stress–strain was studied. Figure 7 shows the simulated stress–strain diagram obtained when Pb_emod, Cb_emod, Ten, and Coh took different values. It can be seen that with the increase in Pb_emod and Cb_emod, the maximum compressive stress increased, while the strain corresponding to the maximum stress decreased. When the values of Pb_emod and Cb_emod exceed 2 × 10^10^ Pa, the stress of the simulated specimen hardly increases, so it can be roughly estimated that Pb_emod and Cb_emod are between 5 × 10^8^ and 2 × 10^10^ Pa. With the increase in Ten and Coh, the maximum compressive stress and the corresponding strain increased. It can be roughly estimated that Ten is between 5 × 10^6^ and 5 × 10^8^ Pa. When Coh is 1 × 10^8^ and 5 × 10^8^, the stress–strain curves of the two specimens coincide, it can be roughly estimated that the Coh is between 5 × 10^6^ and 1 × 10^8^ Pa. At the same time, however, the slope remained unchanged, which means that there was no effect on the compressive modulus of elasticity. Through parameter calibration, the micro-parameters of the two types of concrete were obtained, as shown in Table 4. Figure 8 shows the contact diagram of the specimen, in which 14,207 contacts were generated by the ordinary concrete and 20,310 by the nano-TiO_2_-modified concrete. The parallel effective modulus, tensile strength, and bonding strength of the ordinary concrete were 28.05 GPA, 25 MPa, and 100 MPa, respectively. After 50 dry–wet cycles, the half-particle parallel effective modulus, tensile strength, and bonding strength of the ordinary concrete were 25 GPA, 11 MPa, and 90 MPa, respectively. Meanwhile, the parallel effective modulus, tensile strength, and bonding strength of nano-TiO_2_-modified concrete were 28.05 GPA, 31 MPa, and 100 MPa, respectively. After 50 dry–wet cycles, the half-particle parallel effective modulus, tensile strength, and bonding strength of the nano-TiO_2_-modified concrete were 10 GPa, 17 MPa, and 80 MPa, respectively. This reflects the erosion of concrete caused by the dry–wet cycles, which led to the performance degradation of the particles.

### 4.3. Analysis of the Simulation Results

**Figure 9:**
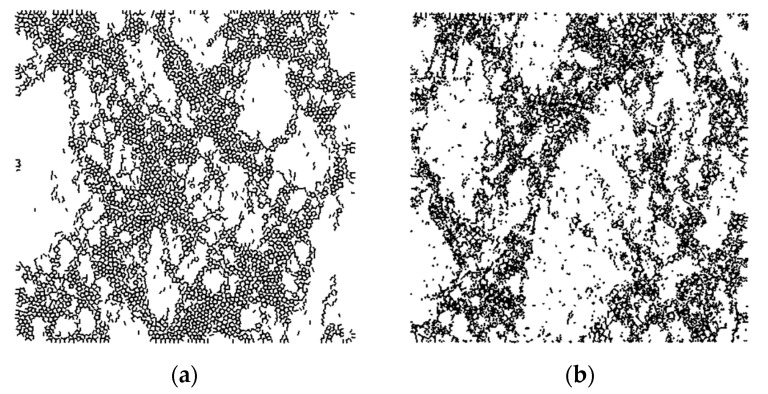
Simulation compression failure diagram of concrete. (**a**) Ordinary concrete. (**b**) Nano-TiO_2_ modified concrete.

**Figure 10:**
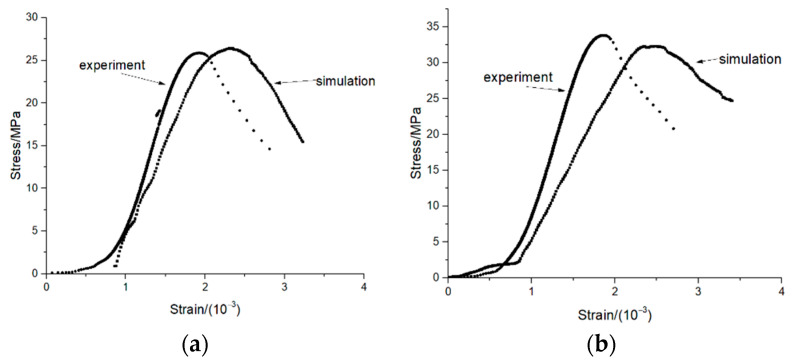
Test and simulated stress–strain diagram of specimens. (**a**) Ordinary concrete. (**b**) Nano-TiO_2_ modified concrete.

Figure 9 shows the crack state of the specimen under compression failure. There are 6841 cracks in ordinary concrete and 8801 cracks in nano-TiO_2_-modified concrete. It can be seen that the number of cracks in the ordinary concrete is more than that in the nano-TiO_2_-modified concrete. Figure 10 shows the comparison between the experimental and simulated stress–strain of specimens. There are some differences in the trend between the simulation and test curves, mainly because the inherent stress–strain trend of the concrete is discrete, so the peak stress and corresponding strain of the specimen were mainly considered in the simulation here. After 50 dry–wet cycles, the peak stress of the ordinary concrete compression test was 25.86 MPa; the corresponding strain was 1.93 × 10^−3^. Meanwhile, the simulated peak stress of the ordinary concrete was 26.4 MPa, and the corresponding strain was 2.27 × 10^−3^. The difference between the test and simulation peak stress was 2.05%, and the corresponding strain difference was 14.98%. Meanwhile, after 50 dry–wet cycles, the peak stress of the nano-TiO_2_-modified concrete compression test was 33.82 MPa, and the corresponding strain was 1.86 × 10^−3^, while the simulated peak stress of the nano-TiO_2_-modified concrete was 32.3 MPa, and the corresponding strain is 2.34 × 10^−3^. The difference between the test and simulation peak stress was 4.71%, and the corresponding strain difference was 20.51%. It can be seen that the simulation materials well reflect the mechanical properties of raw materials, especially the peak compressive strength.

## 5. Conclusions

The main subject of this paper was the influence of nano-TiO_2_ on the resistance of concrete to sulfuric acid attack, including mass loss rate, ultrasonic wave velocity, compressive strength, and XRD analyses. The compression simulation of ordinary concrete and nano-TiO_2_ concrete was carried out by PFC2D software. The result showed that with an increase in the number of dry–wet cycles, the damage to the concrete gradually increased, and adding nano-TiO_2_ improved the sulfate resistance of the concrete. After 50 dry–wet cycles, the mass loss rate of the ordinary concrete was −3.744%, while that of the nano-TiO_2_-modified concrete was −1.363%. The compressive strength of the ordinary concrete decreased by 39.51% from 41.53 to 25.12 MPa, while the compressive strength of the nano-TiO_2_-modified concrete decreased by 35.64% from 49.91 to 32.12 MPa. After the sulfate dry–wet cycles, white crystalline powder was produced on the surface of the nano-TiO_2_-modified concrete, identified as ettringite by XRD analysis. Thus, the simulation materials by PFC2D can well reflect the mechanical properties of raw materials, especially the peak compressive strength.

## Figures and Tables

**Table 1:** Chemical composition of cement.

Component	SiO_2_	Al_2_O_3_	CaO	Fe_2_O_3_	MgO	SO_3_
Cement	22.02	5.2	64.42	5.23	1.02	2.1

**Table 2:** Nano-TiO_2_ properties.

Properties	Density	Melting Point/°C	Boiling Point/°C	Particle Size/nm
Nano-TiO_2_	4.260	1855	2900	25

**Table 3:** Mixture ratio of the nano-TiO_2_-modified concrete.

Group	Cement	Sand	Gravel	Water	Water Reducer	Nano-TiO_2_
Ordinary concrete	415	583	1224	177	4.1	0
Nano-TiO_2_-modified concrete	402.55	583	1224	177	4.1	12.45

**Table 4:** Simulation parameters of compression for specimen.

	Ordinary Concrete	Nano-TiO_2_ Modified Concrete
Minimum radius/mm	0.045	0.01
Maximum radius/mm	0.075	0.075
Normal stiffness/N·m^−1^	1 × 10^9^	1 × 10^9^
Shear stiffness/N·m^−1^	1 × 10^9^	1 × 10^9^
Friction factor	0.577	0.577
Parallel effective modulus/GPa	28.05	28.05
Tensile strength/MPa	25	31
Cohesion/MPa	100	100
Linear effective modulus/GPa	99.33	99.33
Parallel effective modulus after dry–wet/GPa	25	10
Tensile strength after dry–wet/MPa	11	17
Cohesion after dry–wet/MPa	90	80

## Data Availability

Not applicable.
